# Low-cost and reliable substrate-based phenotyping platform for screening salt tolerance of cutting propagation-dependent grass, *paspalum vaginatum*

**DOI:** 10.1186/s13007-024-01225-z

**Published:** 2024-06-19

**Authors:** Zhiwei Liu, Wentao Xue, Qijuan Jiang, Ademola Olufolahan Olaniran, Xiaoxian Zhong

**Affiliations:** 1 National Forage Breeding Innovation Base (JAAS), Nanjing, P. R. China; 2 Institute of Animal Science, Jiangsu Academy of Agricultural Sciences, Nanjing, P. R. China; 3 Key Laboratory for Crop and Animal Integrated Farming of Ministry of Agriculture and Rural Affairs, Nanjing, P. R. China; 4 College of Agriculture, Engineering and Science, University of KwaZulu-Natal, Durban, South Africa; 5 Key Laboratory of Saline-Alkali Soil Improvement and Utilization (Coastal Saline-Alkali Lands), Ministry of Agriculture and Rural Affairs, Nanjing, P.R. China; 6 College of Agro-Grassland Science, Nanjing Agricultural University, Nanjing, P. R. China

**Keywords:** Salt tolerance, Phenotyping system, Average leaf number, Salt_50_, *Paspalum vaginatum*

## Abstract

**Background:**

Salt tolerance in plants is defined as their ability to grow and complete their life cycle under saline conditions. Staple crops have limited salt tolerance, but forage grass can survive in large unexploited saline areas of costal or desert land. However, due to the restriction of self-incompatible fertilization in many grass species, vegetative propagation via stem cuttings is the dominant practice; this is incompatible with current methodologies of salt-tolerance phenotyping, which have been developed for germination-based seedling growth. Therefore, the performance of seedlings from cuttings under salt stress is still fuzzy. Moreover, the morphological traits involved in salt tolerance are still mostly unknown, especially under experimental conditions with varying levels of stress.

**Results:**

To estimate the salt tolerance of cutting propagation-dependent grasses, a reliable and low-cost workflow was established with multiple saline treatments, using *Paspalum vaginatum* as the material and substrate as medium, where cold stratification and selection of stem segments were the two variables used to control for experimental errors. Average leaf number (ALN) was designated as the best criterion for evaluating ion-accumulated salt tolerance. The reliability of ALN was revealed by the consistent results among four *P. vaginatum* genotypes, and three warm-season (pearl millet, sweet sorghum, and wild maize) and four cold-season (barley, oat, rye, and ryegrass) forage cultivars. Dynamic curves simulated by sigmoidal mathematical models were well-depicted for the calculation of the key parameter, Salt_50_. The reliability of the integrated platform was further validated by screening 48 additional recombinants, which were previously generated from a self-fertile mutant of *P. vaginatum*. The genotypes displaying extreme ALN-based Salt_50_ also exhibited variations in biomass and ion content, which not only confirmed the reliability of our phenotyping platform but also the representativeness of the aerial ALN trait for salt tolerance.

**Conclusions:**

Our phenotyping platform is proved to be compatible with estimations in both germination-based and cutting propagation-dependent seedling tolerance under salt stresses. ALN and its derived parameters are prone to overcome the species barriers when comparing salt tolerance of different species together. The accuracy and reliability of the developed phenotyping platform is expected to benefit breeding programs in saline agriculture.

**Supplementary Information:**

The online version contains supplementary material available at 10.1186/s13007-024-01225-z.

## Key message

A novel phenotyping system was developed to screen for salt tolerance of erect stems of *Paspalum vaginatum.*

**Supplementary Information:**

The online version contains supplementary material available at 10.1186/s13007-024-01225-z.

## Introduction

Salinity is a major abiotic stress in agricultural regions, aggravated by large quantities of industrial pollution, excessive use of chemical fertilizers, inappropriate irrigation [1], and more recently, climate change induced by global warming [2, 3]. Therefore, the mechanisms of salt tolerance in plants, involving myriad pathways and genes, remain a top issue for researchers dealing with crop failure under salinization [4]. Crop yields are still limited by high soil salinity [5], despite advances in genomic editing technology [6–9]. A complementary plan for land utilization in coastal or desert saline regions might involve the planting of salt-tolerant grass species for forage production [10].

In the major sexually propagated forage cultivars, such as silo corn [11], sweet sorghum, millet [12], barley, oat, rye, and ryegrass [13], seed germination and seedling growth uniformity can be finely controlled artificially by, for example, seed priming [14] or warm/cold stratification at the initial growth stage [15, 16]. However, forage grasses such as *Paspalum vaginatum*, *Pennisetum purpureum*, and *Miscanthus sacchariflorus* are only weakly self-fertile and their cultivation depends on vegetative propagation, which lacks growth-stage uniformity and subsequently, confounds experimental repeatability.

Seedling tolerance to stresses has been widely investigated in hydroponic systems, which are operationally convenient for controlling salinity, but are disadvantageous for root development since growth in most grass cultivars is aerobic. Moreover, evaporation results in fluctuations in ion concentration, leading to inconsistent stress levels and the need for frequent solution replacements, subsequently raising costs. Alternatively, soil-based potting suits root growth, but lack the ability to control salt concentration, thereby weakening data reliability [17]. These disadvantages have been further revealed by comparing salt tolerance in salt-containing hydroponics and saline soil, where expression of tolerance in the former was not a reliable criterion for the latter [18]. Substrate-based incubation has been successfully applied to phenotype root architecture [19] and frost tolerance [20], and more recently, seedlings under salt stress [21, 22], indicating its potential as a better medium than field soil or sand.

Stress-induced morphological responses (SIMR) at the anatomical level include inhibition of cell elongation, localized stimulation of cell division, and alterations in cell-differentiation status [23]. At the morphological level, responses to salt stress are reflected in plant height and biomass, although the reliability of these indicators is still under debate [18]. Additional scores and scales for growth stages under salt stress have been well-developed for monocotyledons [24], the model plant *Arabidopsis* [25], and barley [26], mainly based on the appearance and number of leaves in the vegetative stage.

Phenotyping of seedlings under salt stress is still subject to methodological limitations [27], where seedling tolerance involves calculating their indices or scores relative to control conditions [18] to minimize the confounding effect of variations among non-stressed seedlings. However, single stress treatments might not be sufficient to extract the comprehensive characteristics of tolerance dynamics under increasing salt concentrations [28], hence affecting both genetic analyses and breeding efforts toward enhanced salt tolerance [27]. On the other hand, sigmoidal mathematical models have been previously applied to describe seed dormancy and germination, as well as germination under salt stress, and key parameters of the simulated curves have been extracted for further genetic analyses [28, 29]. However, the growth dynamics of seedlings derived from cutting propagation are less described by mathematical models, which also have not been adopted for cutting propagation-dependent seedling growth under saline conditions, thus confounding the dissection of major variables controlling their growth behaviors.

## Materials and methods

To develop a low-cost and reliable phenotyping screening platform for not only seed germination-based but also cutting propagation-dependent seedling tolerance under salt stress, in the present study, we compared genotypes of the cutting propagation-dependent grass, *Paspalum vaginatum*, along with seed germination-based warm-season and cold-season grass cultivars for salt stress in a novel system which uses substrate as the medium, selected stem segments as the material, and mathematical simulation curves under multiple stresses as the basis for salt-tolerance estimations. The different comparisons revealed two dominant factors in controlling experimental errors for cutting propagation-dependent grass and a reliable criterion for salt tolerance.

### Genotypes of *P. vaginatum* and warm/cold-season grass cultivars

We used *P. vaginatum* cultivar Adalayd and its related mutagenic offspring SP2, SP3 and SPD1 for salt-tolerance screening, in parallel with warm-season grasses: pearl millet (*Pennisetum glaucum* cv. Wanshu), wild maize (*Purus frumentum* cv. Huafeng3), sweet sorghum (*Sorghum bicolor* cv. Big Kahuna), and cold-season grasses: barley (*Hordeum vulgare* cv. Morex), oat (*Avena sativa* cv. Baiyan2), rye (*Secale cereale* cv. Dongmu70) and ryegrass (*Lolium perenne* cv. Petrel). The phylogeny of *P. vaginatum* is schematized in Additional file 1, where genotypes of SP2 and SP3 are the self-compatible M_1_ generation, and SPD1 is a dwarfed M_2_ individual separated from SP3 seeds. Forty-eight M_2_ recombinants harvested from SP3 were also screened for salt tolerance. All of this material was conserved and provided by the Grass Germplasm Bank of Jiangsu Province, China.

### Workflow of substrate-based phenotyping system for salt-tolerance screening of erect stems of *P. vaginatum*

**Fig. 1:**
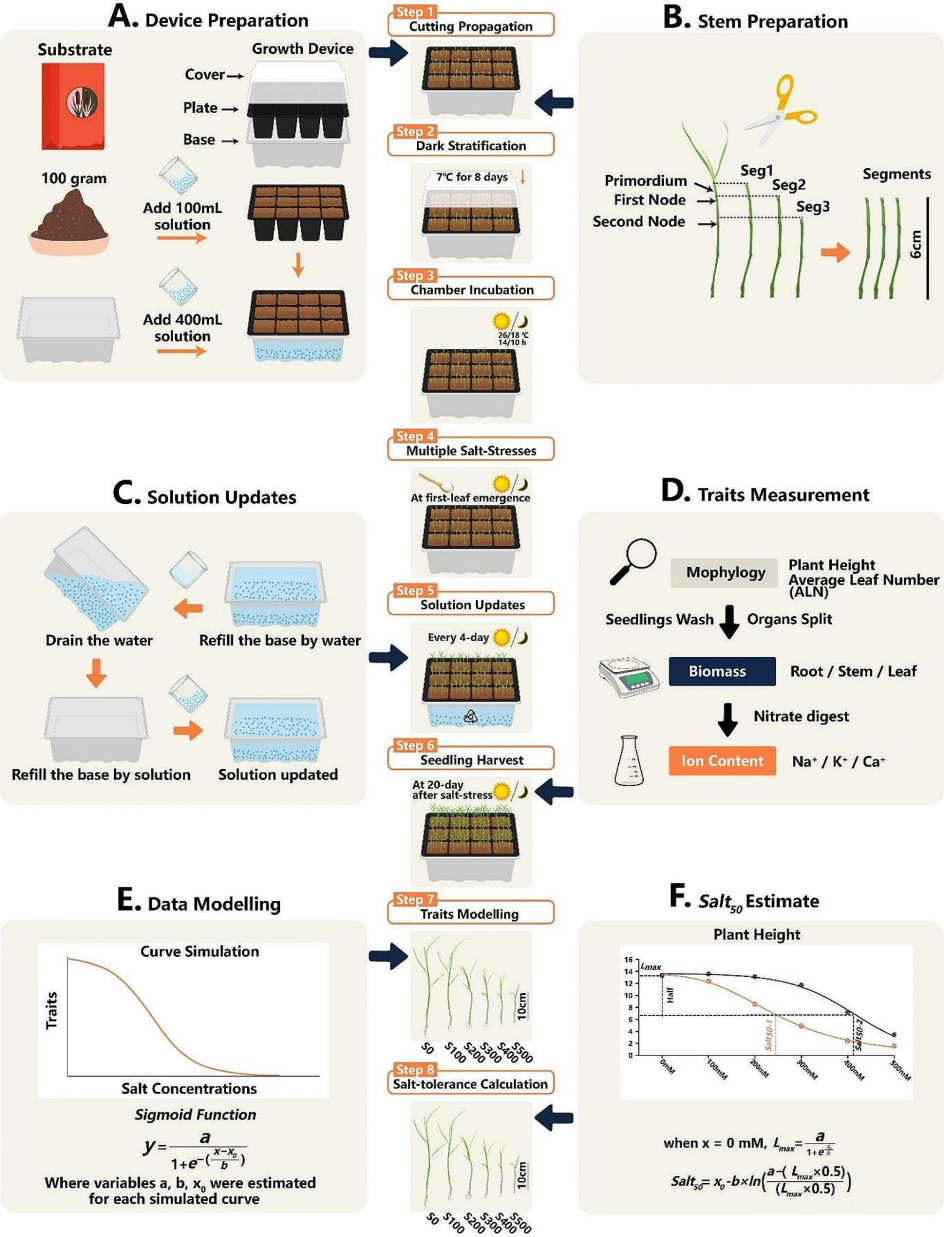
Visualized workflow of substrate-based phenotyping platform for cutting propagation-dependent grasses under salt stress. A 12-hole seedling tray is used to carry the substrate (medium) to maintain the salinity levels (**A**). Erect stems of *P. vaginatum* are prepared in 6-cm segments for cutting propagation (**B**), with Seg2 considered optimal (Step 1). After 8-day stratification at 7 ℃ in the dark (Step 2), the setup (tray, base, and cover) is incubated under day/night conditions of 26/18 ℃ and 14/10 h photoperiod (Step 3). NaCl is applied to the solution in the base at different concentrations when the seedlings reach the first unfolded leaf stage (Step 4). The solution in the base is renewed every 4 days (Step 5 & **C**). Seedlings are harvested at 20 days after treatment for trait measurements (Step 6 &**D**). Data are mathematically modeled by a 3-parameter sigmoid function (Step 7 & **E**), and then parameter-Salt_50_ is calculated according to each simulation curve (Step 8 & **F**)

A seedling tray was used to propagate cuttings of *P. vaginatum* (Fig. 1). Each tray was composed of a base to hold the solution, a 12-hole seedling plate and a transparent cover. Dry soil substrate from Pindstrup Plus Orange (100% blonde peat; 100 g) blended with 100 ml fertilizer solution (Kyle Soluble N-P-K Fertilizer/water = 1/1000) was used to fill each hole in the seedling plate; the plate was then immersed in 400 ml of the same fertilizer solution (Fig. 1A). Once the substrate was saturated, a maximum of 9 erect stems were embedded in each seedling hole dispersively (Fig. 1B, Step 1). The cover was closed and the tray was placed in the dark at 7 ℃ for 8 days of cold stratification (Fig. 1, Step 2), then incubated in a chamber with day/night conditions of 26/18 ℃ and a 14/10 h photoperiod (Fig. 1, Step 3).

Once stem growth reached the first unfolded leaf stage, salt stress was applied (Fig. 1, Step 4). Considering the strong tolerance of *P. vaginatum* to salt stress, we chose 100 mM, 200 mM, 300 mM, 400 mM, and 500 mM NaCl solutions for treatments, in parallel with control conditions (no NaCl added). The solution in the tray base was renewed every 4 days to maintain constant salinities (Fig. 1, Step 5). Specifically, we refilled the base solution to the original weight with water to make up for the water lost by evaporation, then drained the base after 3 h imbibition, and refilled it with the respective solutions to the original weight (Fig. 1C). After 20 days of incubation, seedlings were first measured for morphological traits and then harvested from plates (Fig. 1, Step 6), and rinsed with fresh water for further analysis (Fig. 1D).

Seeds of both warm- and cold-season grass cultivars were similarly added to the surface of the imbibed substrate in the tray as described for *P. vaginatum*, after 30 min surface sterilization (3% hydrogen peroxide). The same cold stratification was also applied to minimize variation among treatments. The cold- and warm-season grasses reached the first unfolded leaf stage after 2 and 4 days, respectively. All grass cultivars germinated fully (> 90%) under our experimental conditions. Salt stress was applied at the one unfolded leaf stage.

In the two-way experimental design, the cold stratification treatment was considered a variable, in parallel with the no-stratification condition. Considering the differences among stem internodes, different intervals of cv. Adalayd’s erect stem were taken as another variable: Segment 1 (Seg1) started upon the primordium apex, Seg2 was clipped upon the first node, and Seg3 was cut upon the second node (Fig. 1B). All three segment types were chopped to 6 cm length, downward from their origins. In the formal workflow, we chose Seg2 as the standard for all experiments, including the validation with the 48 recombinants, and constructed four independent replicates for each *P. vaginatum* genotype.

### Morphological trait and ion content measurements

**Fig. 2:**
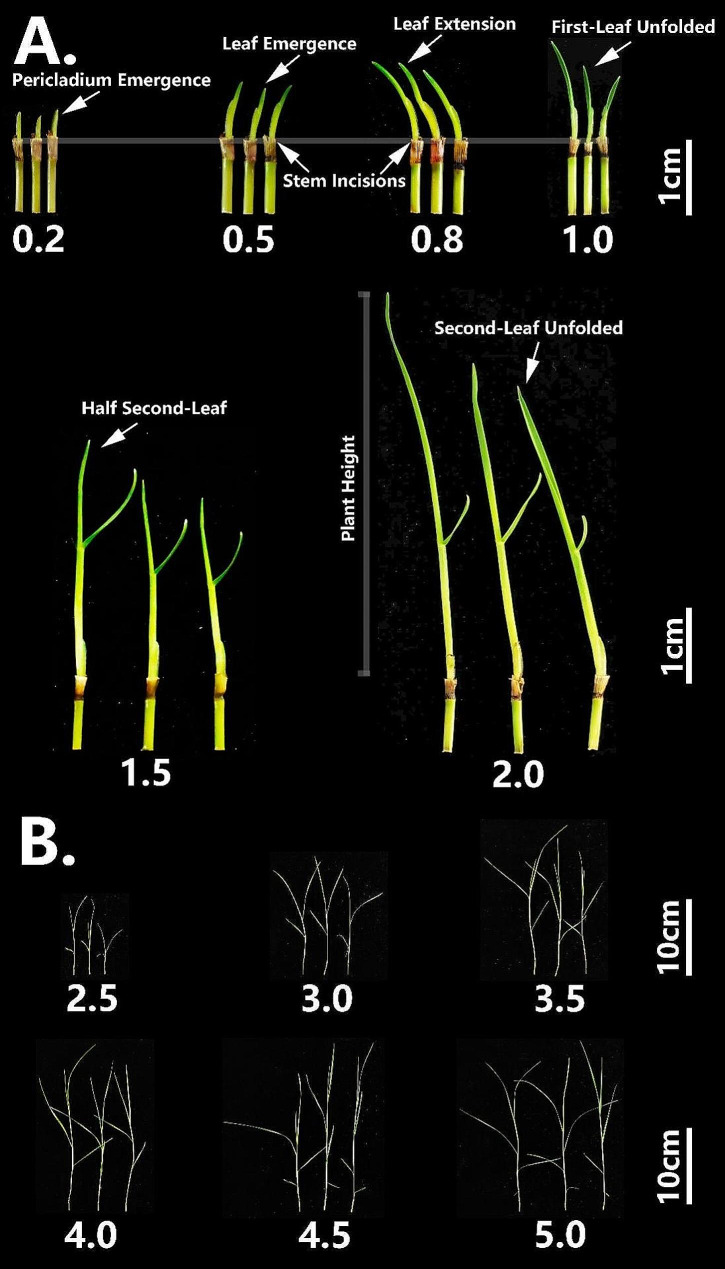
Schematics of digital scale of leaf developmental stage for average leaf number (ALN) estimate. Development of the first-leaf stage is divided into 4 scales (0.2, 0.5, 0.8, 1.0) in (A): pericladium emergence, leaf emergence, leaf extension and leaf unfolding, respectively. Values of 1.5 and 2.0 are used to mark the one-and-a-half leaf and two-leaf stages in (**A**). Accordingly, 2.0, 2.5, 3.0, 3.5, 4.0, 4.5 and 5.0 are photographed in (**B**)

Plant height was measured as the distance from the incision to the growth apex. Leaf developmental stage was also digitalized as average leaf number (ALN) by our established scales which are illustrated in Fig. 2 and described in Table 1. Accordingly, first leaf emergence was divided into four major scales: when the pericladium emerged from the tip of the erect stem, assigned as 0.2; when the first leaf just emerged from the pericladium – 0.5; when the first leaf extended through the pericladium – 0.8; and the first unfolded leaf was assigned the value of 1.0 (Fig. 2A). Using a similar reasoning, the second to fifth leaves were also digitalized (Fig. 2B). In an initial trial with cv. Adalayd, plant height and ALN were measured on selected days after the stress (DAS) was applied: DAS04, DAS08, DAS12, DAS16, DAS20, and DAS24. We selected DAS20 for the formal workflow as the day on which the experiment was terminated.

**Table 1:** Digital scale for average leaf number (ALN) in the substrate-based phenotyping platform, using cutting propagation-dependent grass *P. vaginatum*

ALN Scale	Description
0.2	Pericladium emerged from tip of erect stem
0.5	First leaf just emerged from pericladium
0.8	First leaf extended through pericladium
1.0	First leaf unfolded
1.5	Half second leaf
2.0	Second leaf unfolded
2.5	Half third leaf
3.0	Third leaf unfolded
3.5	Half fourth leaf
4.0	Fourth leaf unfolded
4.5	Half fifth leaf
5.0	Fifth leaf unfolded

The seedlings were harvested and root, stem, and leaf organs were separated manually and oven-dried for biomass measurements. Dry samples were further digested with 5 ml nitrate on a block digestion system (PerkinElmer SPB50-48) for 10 min at 70 ℃, 40 min at 130 ℃, and nitrate dry-out at 160 ℃, and 25 ml volumetric solution was filtered through a syringe (pore size = 0.45 μm). Na^+^, K^+^ and Ca^2+^ concentrations were measured by ICP-OES (PerkinElmer Avio200) and the corresponding ion contents were calculated by standard curves with ICP Standard Solution (Agilent, ICM-462).

To measure the ion stability in the solution contained in the base, 2 ml was sampled into a digestion tube from each box after replenishing the solution with water, then dehydrated at 90 ℃. Following the above digestion procedures, 5 ml nitrate was added to the tube. Final ICP-OES intensities of Na^+^ were calibrated by the values measured from standard solutions of 0, 100, 200, 300, 400, and 500 mM NaCl, and calculated into equivalent concentrations. The K^+^ content was only measured by ICP intensities.

### Mathematical model simulations for curve dynamics and salt-tolerance calculations

Mathematical modeling of seedling growth under multiple salt concentrations was conducted as previously described [29] and as illustrated in Fig. 1E, with curves simulated by the 3-parameter sigmoid function, which best fit the six experimental observations compared to the other functions:

1 $$y = \frac{a}{{1 + {e^{ - \left( {\frac{{x - {x_0}}}{b}} \right)}}}}$$

where variables *a*, *b* and *x*_*0*_ were estimated by Sigmaplot 14.0 and defined as follows: *a* is a limit value for *Y*_*max*_, *b* controls the shape and steepness of the simulated curve, and *x*_*0*_ is the half-maximal activation level of the curve. *Y*_*max*_ was calculated for each genotype by Eq. (1) as follows:

1 $${\rm{when}}\,x = \,0\,mM,\,{Y_{max}} = \frac{a}{{1 + {e^{\frac{{{x_0}}}{b}}}}}$$

Salt_50_ represents the NaCl concentration (*x* axis) at which the seedling *Y*_*max*_ is reduced by half (Fig. 1F), and was calculated by solving Eq. (2) as follows:

2 $$Sal{t_{50}}\left( {{\rm{mM}}} \right) = {x_0} - b \times ln(\frac{{a - \left( {{Y_{max}} \times 0.5} \right)}}{{\left( {{Y_{max}} \times 0.5} \right)}})$$

The data for each treatment were averaged for further curve simulation for all of the experimental material. Two-way ANOVA was conducted using STATISTICA 7, taking stratification and segments as factors. A principal component analysis (PCA) was run with all of the collected phenotypic data using the software PAST, and graphed with GraphPad Prism 10. Pearson correlation matrix was calculated by R package “corrplot” at a significance level of *p* < 0.005.

## Results

### Two major experimental variables determining seedling performance of cutting propagation-dependent grass under salt stress

**Fig. 3:**
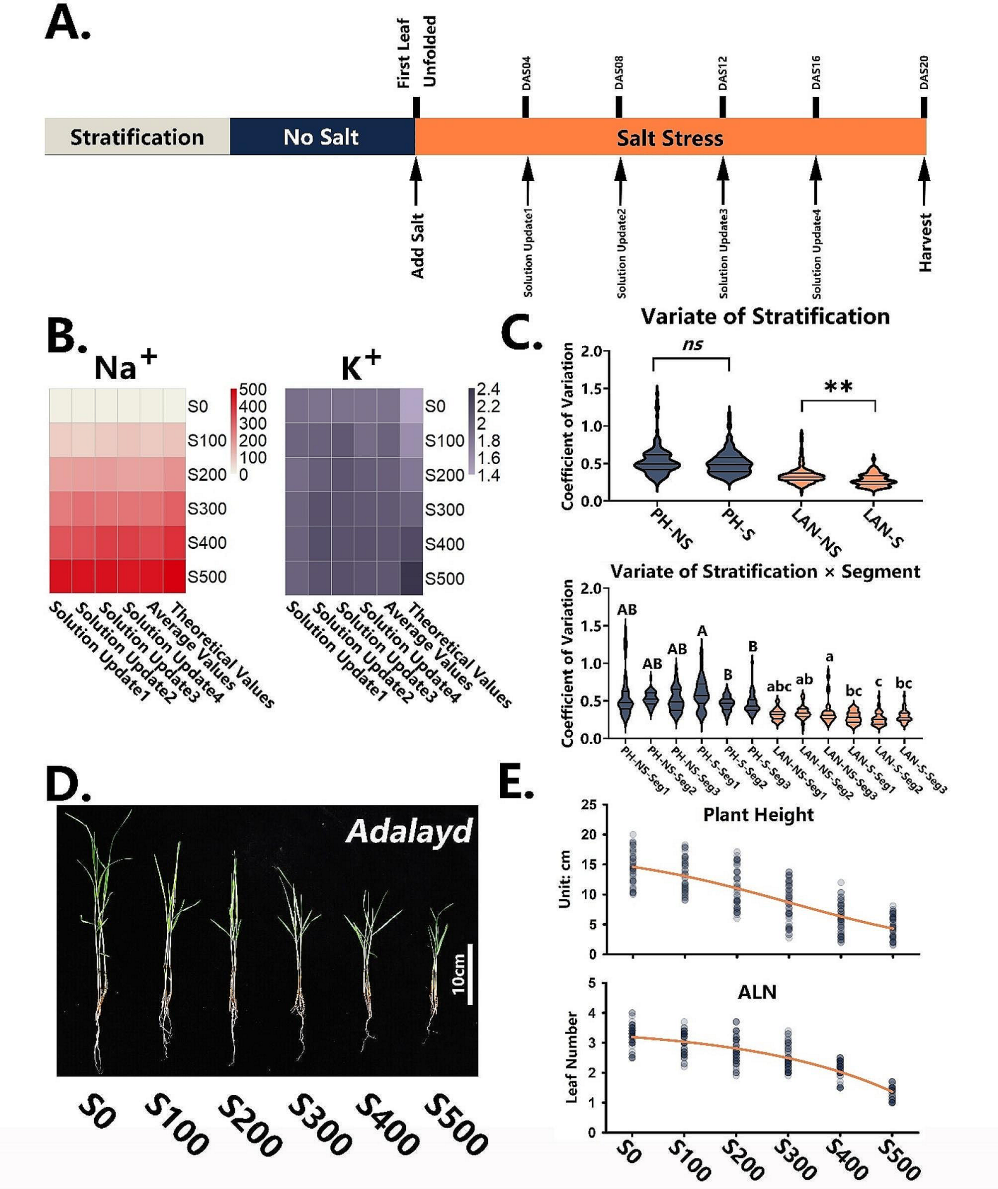
Stepwise validation of major factors determining experimental errors in seedling performance of cutting propagation-dependent grass under salt stress. (**A**) The overall procedure. (**B**) Na^+^ and K^+^ concentrations at each solution renewal step. Squares in Na^+^ heatmap represent concentrations, and in K^+^ heatmap, ICP intensities. (**C**) Comparison of CVs of plant height (PH) and average leaf number (ALN) for stratification (S = stratification, NS = no stratification), and segment factor (Seg1 = segment1, Seg2 = segment2, Seg3 = segment3); ns, not significant; **p < 0.01 (t-test); different letters indicate significant difference at *p* < 0.05 (Tukey HSD); uppercase and lowercase letters represent two independent comparisons. (**D**) Final seedling stems of cv. Adalayd after different treatments (S0 = no salt; S100, S200, S300, S400, S500 = 100, 200, 300, 400 and 500 mM NaCl, respectively) are shown; bar = 10 cm. (**E**) PH and ALN values of each replicate are plotted against the salinities and fitted with the 3-parameter sigmoid function for dynamic curves

Four *P. vaginatum* genotypes, and three warm-season and four cold-season grass cultivars were compared for salt tolerance using our substrate-based salt-stress phenotyping protocol, illustrated in Fig. 3A. At the first unfolded leaf stage, different amounts of NaCl were added to the solution in the container base to simulate different levels of salt stress. The solution was renewed every 4 days. To verify constant solution salinities during the entire experimental stress period, solutions of each NaCl concentration were sampled at each analyzed time point for Na^+^ and K^+^ contents (Fig. 3B). Both Na^+^ and K^+^ presented stable concentrations at all time points, confirming a reliable stress strength and adequate nutritional conditions, respectively.

Preliminary trials revealed that in contrast to seed germination, growth uniformity of the cuttings propagated from the erect stem stage was not significantly related to growth conditions (data not shown). Here we considered stratification (S)/no stratification (NS), and segments (Seg1, 2, 3; Fig. 1B) as the two major variables determining the experimental errors for stem growth after cutting propagation. The coefficients of variation (CVs) for both plant height and ALN were further tested. Plant height following S or NS showed a non-significant difference (*p* = 0.5707), but the CVs of ALN after stratification vs. no stratification were significantly lower (*p* = 0.0085, Fig. 3C). In the crossed experimental design, stratification combined with Seg2 gave the lowest CVs for both plant height and ALN (Fig. 3C).

Two-way ANOVA (Additional file 2) displayed sum of squares (SS) of 86.4% and 64.5% for the stratification and segment parameters corresponding to ALN and plant height, respectively, exhibiting the dominance of stratification for the ALN trait but that of segment for plant height. The statistics suggested divided responses of the morphological traits to the different experimental variables when estimating the uniformity of seedling performance under multiple salinities.

Moreover, the duration of seedling incubation was also examined by comparing six time points: DAS04, DAS08, DAS12, DAS16, DAS20, and DAS24. Plant height and ALN were respectively investigated at each time point, and further simulated by the sigmoidal function. The R^2^ coefficients of the curve simulations were compared (Additional file 3). At DAS20, coefficients of the simulated curves for both morphological traits were well saturated at R^2^ > 0.98, meaning that 20 days of incubation was sufficient.

We validated full procedures on *P. vaginatum* cv. Adalayd, setting up the conditions of cold stratification, Seg2 stem, and 20 days incubation after salt stress as the formal parameters, and the final seedling propagation from erect stems is shown in Fig. 3D. Values of plant height and ALN vs. salt concentration were plotted (Fig. 3E) and gave dynamic sigmoid and parabola-shaped curves, respectively, suggesting their different responses to salinity.

### Profiling morphological dynamics under multiple salinities reveals the reliable criterion of salt tolerance

**Fig. 4:**
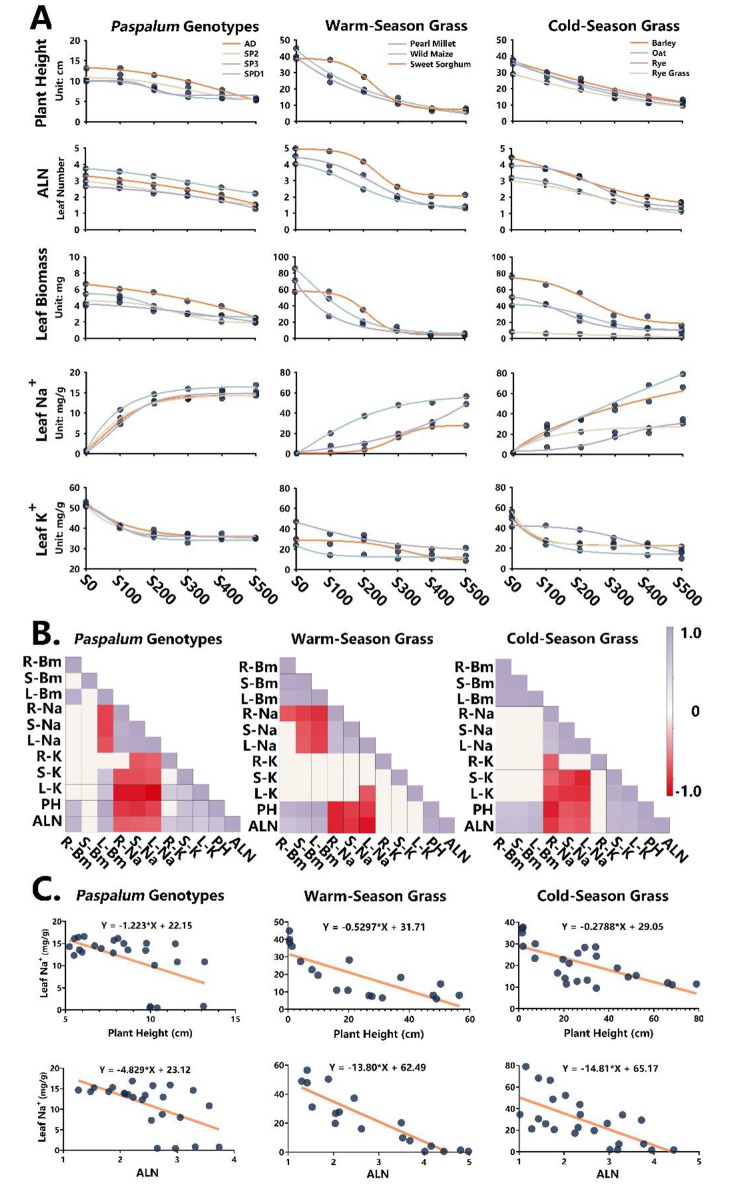
Trait dynamics vs. salinities and trait correlations among *P. vaginatum* genotypes, and warm-season and cold-season grass cultivars. (**A**) Plant height, average leaf number (ALN), and leaf biomass and Na^+^ and K^+^ contents are plotted against salinity level using the 3-parameter sigmoid function for the curve simulations. S0 = no salt; S100, S200, S300, S400, S500 = 100, 200, 300, 400 and 500 mM NaCl, respectively. (**B**) Correlation matrix of 11 traits constructed by the Pearson method; significant correlation coefficients at *p* < 0.005. Purple and red color refer to positive and negative correlations, respectively. PH = plant height, Bm = biomass, R = root, S = stem, L = leaf. (**C**) Linear regression analysis of the relationship between leaf Na^+^ content and traits of PH/ALN is performed with the listed regression equations

The morphological traits plant height, ALN, biomass, and Na^+^, K^+^ and Ca^2+^ contents of each seedling organ were plotted against salinity: 0, 100, 200, 300, 400 and 500 mM NaCl (Fig. 4A and Additional file 4). Datasets for most of the traits were well-simulated by sigmoidal curves, except stem biomass in *P. vaginatum* genotypes and Ca^2+^ content in all material (shown in Additional file 4). In general, all of the traits decreased with increasing salinity (Fig. 4A), as a consequence of the Na^+^ accumulation.

Overall, morphological traits (plant height, ALN, and leaf biomass) of the warm-season and cold-season grass cultivars exhibited steep curves in response to increasing salt strength, in contrast to the flatter trend of the *P. vaginatum* genotypes. Notably, the average leaf Na^+^ content in *P. vaginatum* genotypes increased sharply to a maximum of 15 mg/g, compared to the drastic accumulation to 40–90 mg/g in grass cultivars except sweet sorghum and ryegrass. In contrast, leaf K^+^ contents decreased to an average level of 20 mg/g in all grass cultivars, compared to the abundant K^+^ resource (40 mg/g) in *P. vaginatum* genotypes.

Looking at the individual plant types, cv. Adalayd gave the highest values of plant height and biomass but the lowest level for leaf Na^+^ content, in contrast to the pattern of the SPD1 genotype. ALN of SPD1 was significantly higher than in other genotypes among the salt-stress treatments. In the category of warm-season grasses, sweet sorghum stood out mainly due to the distinct sigmoid curves for both morphological and ion traits. Similarly, ryegrass showed unique trends in all traits compared to the other cold-season grasses.

To discover the best criterion for tolerance to accumulated ions, a Pearson correlation matrix with significance at *p* < 0.005 was conducted for the *P. vaginatum* genotypes, and the warm-season and cold-season grass cultivars (Fig. 4B). Aside from the strong correlations displayed within groups for biomass and ion contents, plant height and ALN were both strongly negatively correlated with Na^+^ contents of all organs. In total, 21 and 22 correlations beyond the significance threshold were achieved for plant height and ALN in all three matrixes. To further confirm the relationships between leaf Na^+^ contents and plant height/ALN, linear regression analysis was performed with the regression equations in Fig. 4C, resulting in robust negative dependencies, and indicating accurate representations of plant height and ALN for leaf Na^+^ contents under salt stress. Therefore, we regarded both plant height and ALN as the “aerial” traits correlated to ion accumulation and salt tolerance.

### Key parameter of simulated curve can screen for *P. vaginatum* genotypes inheriting extreme tolerance to salinity

**Fig. 5:**
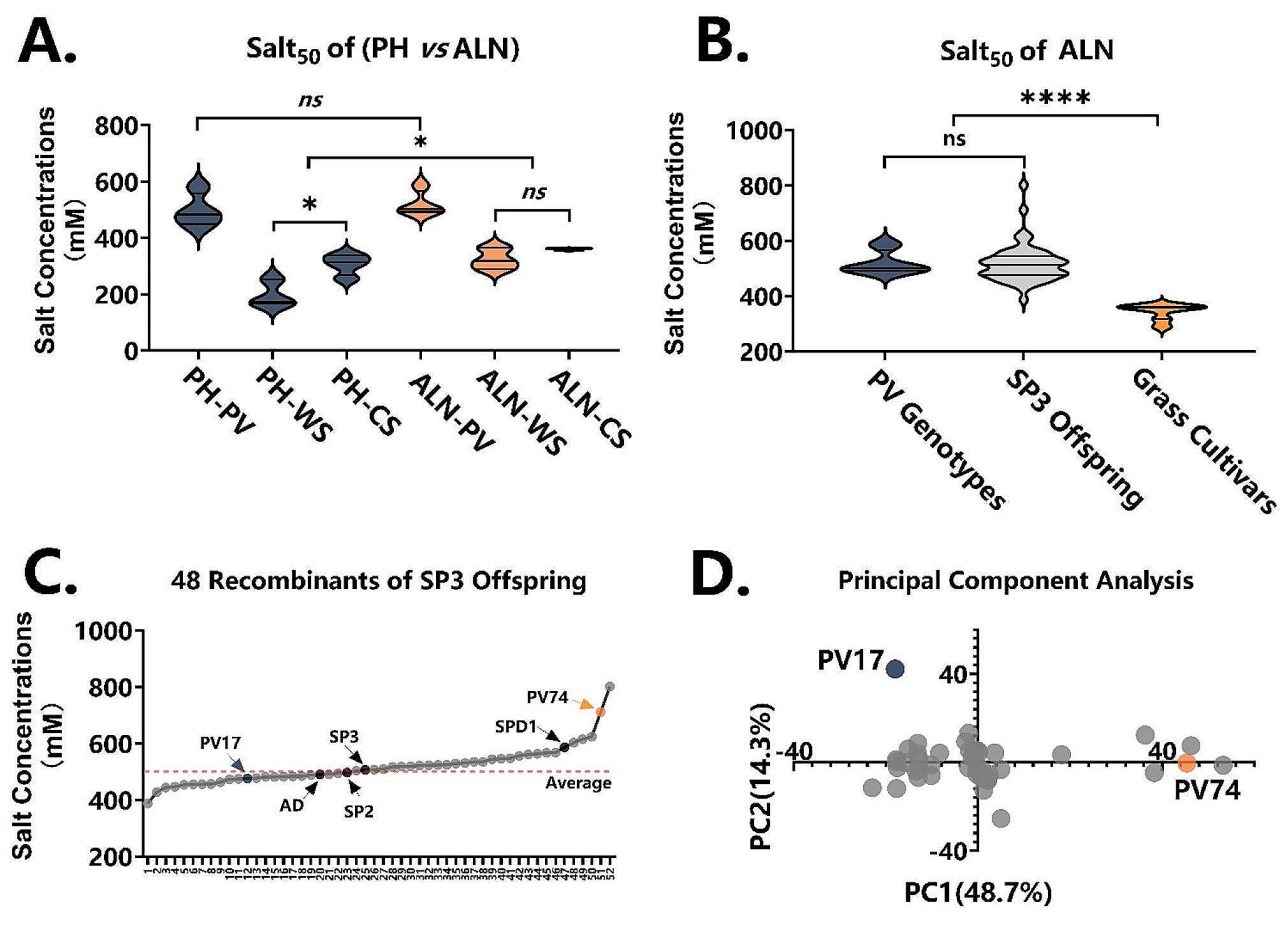
Salt tolerance estimation for different categories of grass cultivars and recombinants of *P. vaginatum* offspring. (**A**) The key parameter of Salt_50_, representing salt tolerance, is calculated according to simulated curves of plant height (PH) and average leaf number (ALN), and compared among *P. vaginatum* genotypes (PV), and warm-season (WS) and cold-season (CS) grass cultivars; statistical significance is shown. (**B**) 48 recombinants of SP3 offspring combined with *P. vaginatum* genotypes and grass cultivars (warm- and cold-season cultivars). (**C**) Salt_50_ values for each *P. vaginatum* genotype and 48 recombinants (52 in total); 4 *P. vaginatum* genotypes, and the extreme lines PV17 and PV74, are indicated with arrows. (D) PCA performed on 48 recombinants, using data of biomass and ion content

We used Salt_50_ as the key parameter of the simulated curves to estimate the salt tolerance of the different genotypes and cultivars. The two aerial traits, plant height and ALN, were assigned for their Salt_50_ calculations, and the results are compared in Fig. 5A. For *P. vaginatum* genotypes, no significant differences were detected between plant height-Salt_50_ and ALN-Salt_50_. However, the Salt_50_ values of warm- and cold-season grasses calculated from plant height were clearly lower than those calculated from ALN. More specifically, the Salt_50_ parameters varied significantly between warm- and cold-season grasses when calculated from plant height, but insignificantly when calculated from ALN. It seems that the plant height-derived Salt_50_ values are divided around the general threshold of 300 mM for most of the grass cultivars, but ALN-based parameters narrowed the variation among different species.

Taking the lower CVs in the seedling replicates (Fig. 3C) and equivalent data range on *y* axis among various species (Fig. 4A) as priors, we applied ALN-dependent Salt_50_ for further salt tolerance estimations. Using the aforedescribed workflow, 48 recombinants were randomly selected from a seed population of SP3 offspring to screen for salt tolerance. The Salt_50_ calculated from their ALN curves are grouped in Fig. 5B, and further arranged in ascending order by individuals (Fig. 5C). An average Salt_50_ value of 500 mM was observed for the group of SP3 offspring, which is much higher than the average of 350 mM in the grass cultivars. Three genotypes of *P. vaginatum* were ordered in a middle position of the SP3 seed population, leaving the SPD1 ranked in the extreme high level of salt tolerance (Fig. 5C). Moreover, as indicated by the line extremities in Fig. 5C, recombinants of PV74 and PV17 were also highlighted in the PCA plot (Fig. 5D), which was constructed from plant height/ALN-independent datasets of organ biomass and Na^+^ and K^+^ contents. This confirmed that the key parameter Salt_50_ calculated from ALN-simulation curves is valid for salt tolerance screening of populations with big sample sizes.

## Discussion

### Substrate-based platform for salt stress application as an intermediate between hydroponic cultivation and potting

To maintain a stable pH environment, hydroponic solutions have to be renewed every 2 days in salt-stress studies, as found with rice [30], barley [31], wheat [32], and cucumber [33], and equipped with an air pump for sufficient oxygen supplementation. Strong correlation matrices can be achieved between root and shoot traits under these elaborate conditions [33]. To simplify the operational conditions, soil-like substrate has recently become popular in potting experiments for short salt-stress incubation, e.g., with maize seedlings, but abundant gene functions still have to be verified [21, 22, 34], even though the actual salt concentration in the substrate is irrelevant after the one-time watering to saturation. This might limit long-term screening results, especially at low salinity levels, because most of the salt is gradually taken up by the plants.

In the present study, this same substrate was used as the medium in a 12-hole seedling tray with a base holding the corresponding solution (Fig. 1A). The substrate in the plate easily absorbs the salt and other nutrients present in the solution in the base, and the tray can be separated from the base as well—a great convenience for solution renewal (Fig. 1C). Indeed, measurements of the solution in the base confirmed stable concentrations of both Na^+^ and K^+^ with renewal every 4 days (Fig. 3B). The small granular structure of the organic substrate prevents leaching from the plate and subsequently maintains strong buffering power in terms of salinities; it also creates more space between the granules, benefiting root development in comparison to pure hydroponics. In fact, substrate–solution mixtures tend to provide an intermediate status between pure hydroponics and substrate-based potting; they not only maintain a stable level of stress but are also suitable for root growth. Therefore, our use of the seedling tray and base with substrate provided stable stress levels and sufficient interspacing for root development, with no need for sophisticated procedures, thus improving experimental operability.

### Salt stress in cutting propagation is distinct from that in seed germination

Cold stratification is frequently used to obtain uniform seedling performance in vernalization-requiring plants, such as *Arabidopsis* [35], most of the winter-type Triticeae [36, 37], and some grass species [38]. However, due to its weak ability to produce seeds (except cv. Sea Spray), the flowering time of *P. vaginatum* is of less concern [39]. From our observations, vernalization could promote *P. vaginatum* growth and early flowering. Hence, it is not surprising that the seedling emergence from *P. vaginatum* erect stems was significantly unified by the 8-day cold treatment, as reflected in the comparison of ALN-NS and ALN-S groups (Fig. 3C); moreover, stratification tended to be the dominant factor in controlling CVs of ALN (two-way ANOVA, Additional file 2), a novel result.

Surprisingly, Seg2 was the best stem fragment for cutting propagation under salt stress, regardless of the application of stratification (Fig. 3C). This implies that the seedling performance of erect stems is not gradually improved by lower intervals, as evidenced by fact that the CVs of ALN-S-Seg3 were not significantly reduced compared to those of Seg2. The variations caused by stem segments also could not be narrowed by improving growing conditions (data not shown). This phenomenon is probably due to the different physiological functions of stem internodes in grass, as determined genetically for sorghum [40]. The internode between the primordium and first node is believed to be the growth apex for leaf formation; its growth rate mainly up to the primordium status at sampling time is therefore dramatically altered, as is that of the internode between the second and third node, which might be responsible for stem elongation. All of these situations complicate stem cutting propagation more than do effects of seed germination.

Moreover, the dynamics of leaf Na^+^ content in stem propagation could be distinguished (Fig. 4A), characterized by the steep increase in Na^+^ content in the 100 mM NaCl treatment among all 4 *P. vaginatum* genotypes, in contrast to initial Na^+^ increases in other grass cultivars corresponding to low salinity. It seems that leaf Na^+^ accumulation occurs without any retention at low salinities in *P. vaginatum* genotypes, but somehow stagnates at a stable level (15 mg/g) responding to the salinity increases (Fig. 4A). Here we hypothesized that the stem might take up Na^+^ through the incisions at the early stage of cutting propagation due to the delayed root emergence, which also might limit Na^+^ transport under high salinities. Unlikely, Na^+^ uptake in cold/warm-season grass must be managed by the root, which is initiated first during seed germination then restricts Na^+^ transport. These points require further confirmation.

### ALN and it derived parameter is more suitable for profiling stress-induced morphological responses

Plant height and biomass are easily disturbed by many subtle factors. For instance, as we discussed above, the stem fragments carrying the primordium or elongation internodes can hardly be unified for repeatability, and this certainly hinders the phenotyping of salt tolerance. This issue is reflected not only in a previous study on soybean tolerance to salt stress [41], but also in the two-way ANOVA results showing segment as a dominant factor for CVs in plant height (Additional file 2). Biomass is also frequently affected by leaf shape and size, as reported in cucumber [33] and other crops [42].

Moreover, these morphological traits do not contain the core feature of stress-induced morphological responses, especially the aspect of alterations in cell differentiation, but are more inclined to be useful for selection through breeding. In contrast, leaf number tends to reflect the actual developmental stage under both non-stress [43] and salt-stress [44] conditions, which might not be significantly disturbed by variations in meristem potential and organ shape. Based on this advantage, the dynamics of salt tolerance depicted by ALN is reflected in the most uniform curves in the comparisons of Fig. 4A and Additional file 4, considering that both axes are calibrated on the same scale. It is prone to overcome the barriers when comparing salt tolerance of different species together.

However, the ALN-based parameter displayed a higher Salt_50_ than the plant height-derived Salt_50_, challenging the average threshold of salt tolerance at around 300 mM [45]. Nevertheless, significance testing for plant height-Salt_50_ between warm- and cold-season grass cultivars indicated significantly lower salt tolerance in the former; this was not fully supported by our results since only the root Na^+^ content of cold-season grasses was higher (Additional file 4), nor has it been sufficiently reported in the literature. This discrepancy might arise from the faster growth rate of warm-season grasses, resulting in higher plant height for the no-salt treatment under our incubation conditions; this does not occur with the ALN-based parameter. Moreover, ALN-based salt tolerance was validated by 48 recombinants of SP3 offspring; the extreme lines (PV17 and PV74) for ALN-Salt_50_ were consistently divergent in the PCA plot (Fig. 5C, D) using completely different datasets. Overall, ALN-derived Salt_50_ might more realistically reflect salt tolerance.

### Halophyte or glycophyte? *P. vaginatum*, a potential model plant for salt-tolerance studies

Designated as a halophyte, the strong tolerance of *P. vaginatum* to high salinities has been well-established [46–48]. Based on the average of Salt_50_ calculated from the 48 recombinants, almost 500 mM NaCl solution is required to halve its leaf numbers, nearly on par with a number of halophytes if considering only the tolerated concentrations, including *Thellungiella halophila* (500 mM) [49], *Plantago crassifolia* (400 mM) [50], *Puccinellia tenuiflora* (400 mM) [51], and *Suaeda maritima* (400 mM) [52], as recently summarized [3]. However, distinct from the preference for high Na^+^ accumulation in halophytes (higher than 45 mg/g) [53], low Na^+^ concentrations were detected not only within our *P. vaginatum* genotypes (below 15 mg/g) but also in others (below 9 mg/g) [46], raising the question of whether *P. vaginatum* should be regarded as a type of halophyte.

Definitions of halophytes are still manifold [54] in the context of salt-tolerant plants defined as ‘obligatory’ or ‘facultative’ halophytes [3]. Halophytes are believed to have begun as wild plants adapted to saline environments that were able to survive and complete their life cycle in habitats with a soil salinity equivalent to at least seawater (from our understanding). Advances in our knowledge are key to really understanding halophyte biology. Even so, typical halophytes have several similarities, one being active salt uptake under low-salt conditions, implying the existence of inherent mechanisms of constitutive stress defense and homeostasis [55]. Our results displayed active uptake of Na^+^, but mainly due to stem transport in the process of cutting propagation (as discussed above). It seems that *P. vaginatum* is able to avoid salt stress by preventing Na^+^ import, which better fits the halotropism model of glycophytes [56, 57]. We therefore prefer to regard *P. vaginatum* as a strongly salt-tolerant glycophyte turfgrass. Compared to the distant wild halophytes, the mechanism in *P. vaginatum* for shutting Na^+^ out of its organs might be more constructive for salt-tolerance improvement in staple crops.

To conclude, a low-cost and reliable phenotyping platform is introduced for screening not only seed germination-based but also cutting propagation-dependent seedlings’ tolerance to various levels of salt stress. *P. vaginatum* genotypes, and warm-season and cold-season grass cultivars were compared using this platform, taking substrate as the medium and mathematical simulation curves as the basis for salt-tolerance estimations. The procedure of cold stratification and selection of stem segments were considered two principals in controlling experimental errors. Among morphological traits, ALN and its derived parameter Salt_50_ were designated as the best criteria for evaluating salt tolerance. The integrated platform was also further tested by screening 48 recombinants, where the genotypes displaying extreme ALN-based salt tolerance were also highlighted in the datasets of biomass and ion content, confirming the reliability of our phenotyping platform, as well as the reproducibility of ALN as an indicator of salt-tolerance. The accuracy and reliability of this phenotyping platform is expected to benefit breeding programs in saline agriculture.

### Electronic supplementary material

Below is the link to the electronic supplementary material.

Supplementary Material 1

Supplementary Material 2

Supplementary Material 3

Supplementary Material 4

## Data Availability

The datasets used and/or analyzed during the current study are available from the corresponding authors on reasonable request.
