# Supplementary material for: Low-cost and reliable substrate-based phenotyping platform for screening salt tolerance of cutting propagation-dependent grass, paspalum vaginatum
Source: Plant Methods. 2024 Jun 19;20:94. doi: 10.1186/s13007-024-01225-z (PMC11186238; doi:10.1186/s13007-024-01225-z)
Supplement: Supplementary file 1 — Supplementary Material 1 [file 13007_2024_1225_MOESM1_ESM.docx]

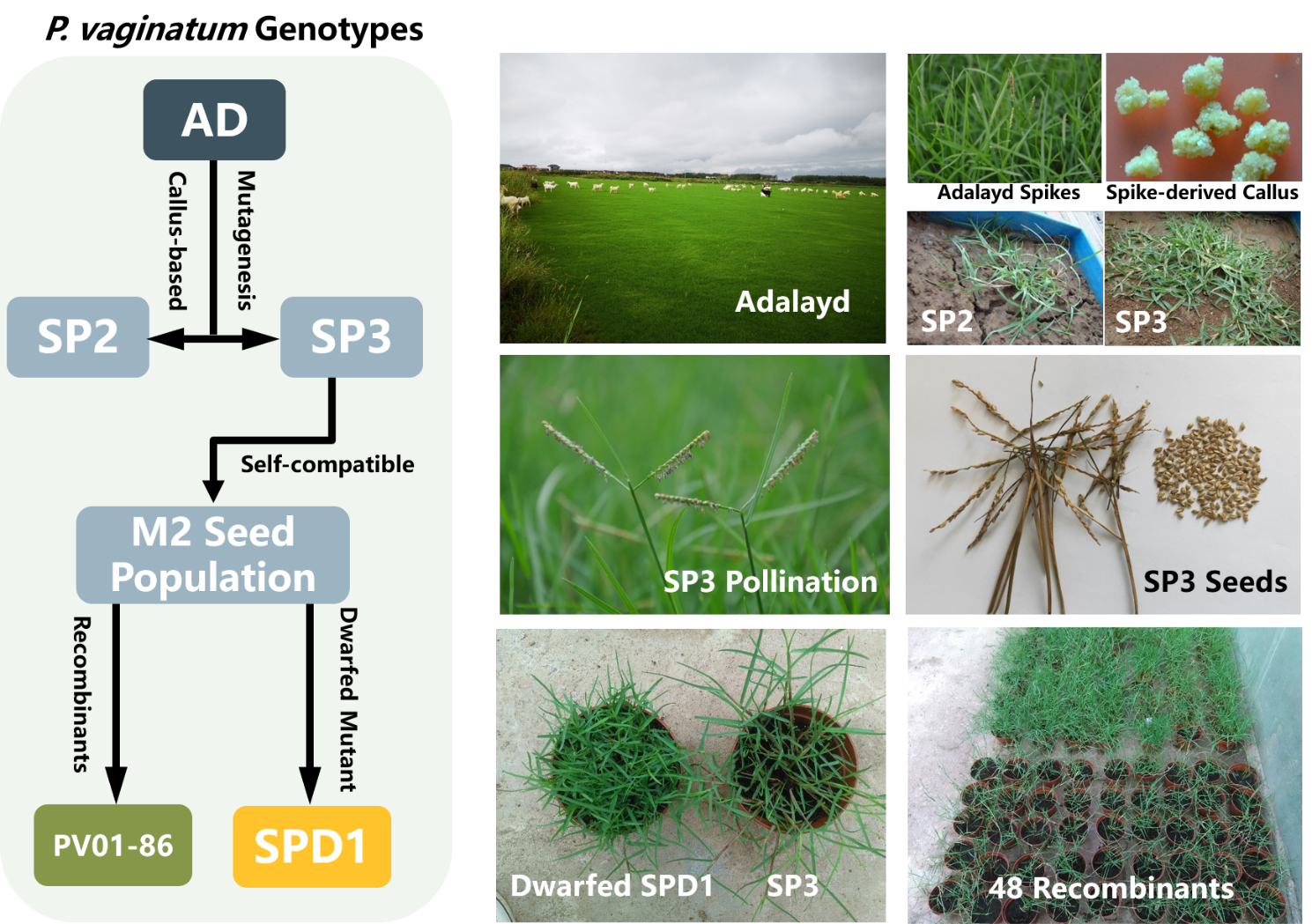


**Additional file 1. Phylogeny of *P. vaginatum* genotypes.** Young spikes of cv. Adalayd were first used to generate the callus, then mutagenized to obtain the self-compatible M_1_ generations, SP2 and SP3. M_2_ seed population was obtained from the self-fertile SP3, and a dwarfed offspring, SPD1, was further screened out. An additional 48 recombinants were also randomly selected for salt-tolerance estimations.
