# Supplementary material for: Low-cost and reliable substrate-based phenotyping platform for screening salt tolerance of cutting propagation-dependent grass, paspalum vaginatum
Source: Plant Methods. 2024 Jun 19;20:94. doi: 10.1186/s13007-024-01225-z (PMC11186238; doi:10.1186/s13007-024-01225-z)
Supplement: Supplementary file 2 — Supplementary Material 2 [file 13007_2024_1225_MOESM2_ESM.docx]

**Additional file 2. Two-way ANOVA on CVs of plant height (PH) and average leaf number (ALN) measured in two-factor experimental design.**

|  | Stratification (S) | | Segment (Seg) | | S × Seg | | Error | Total |
| --- | --- | --- | --- | --- | --- | --- | --- | --- |
|  | SS | % ^a^ | SS | % ^a^ | SS | % ^a^ | SS | SS |
| DF | 1 | | 2 | | 2 | | 246 | 251 |
| PH | 0.0536 | 13.9% | 0.2490** | 64.5% | 0.0515 | 13.3% | 0.0320 | 0.3861 |
| ALN | 0.2550** | 86.4% | 0.0077 | 2.6% | 0.0217 | 7.3% | 0.0109 | 0.2953 |

^a^ Percentage of SS (sum of squares) was calculated by dividing by total SS.

**Significant differences at *p* < 0.001.
