# Supplementary material for: Low-cost and reliable substrate-based phenotyping platform for screening salt tolerance of cutting propagation-dependent grass, paspalum vaginatum
Source: Plant Methods. 2024 Jun 19;20:94. doi: 10.1186/s13007-024-01225-z (PMC11186238; doi:10.1186/s13007-024-01225-z)
Supplement: Supplementary file 3 — Supplementary Material 3 [file 13007_2024_1225_MOESM3_ESM.docx]

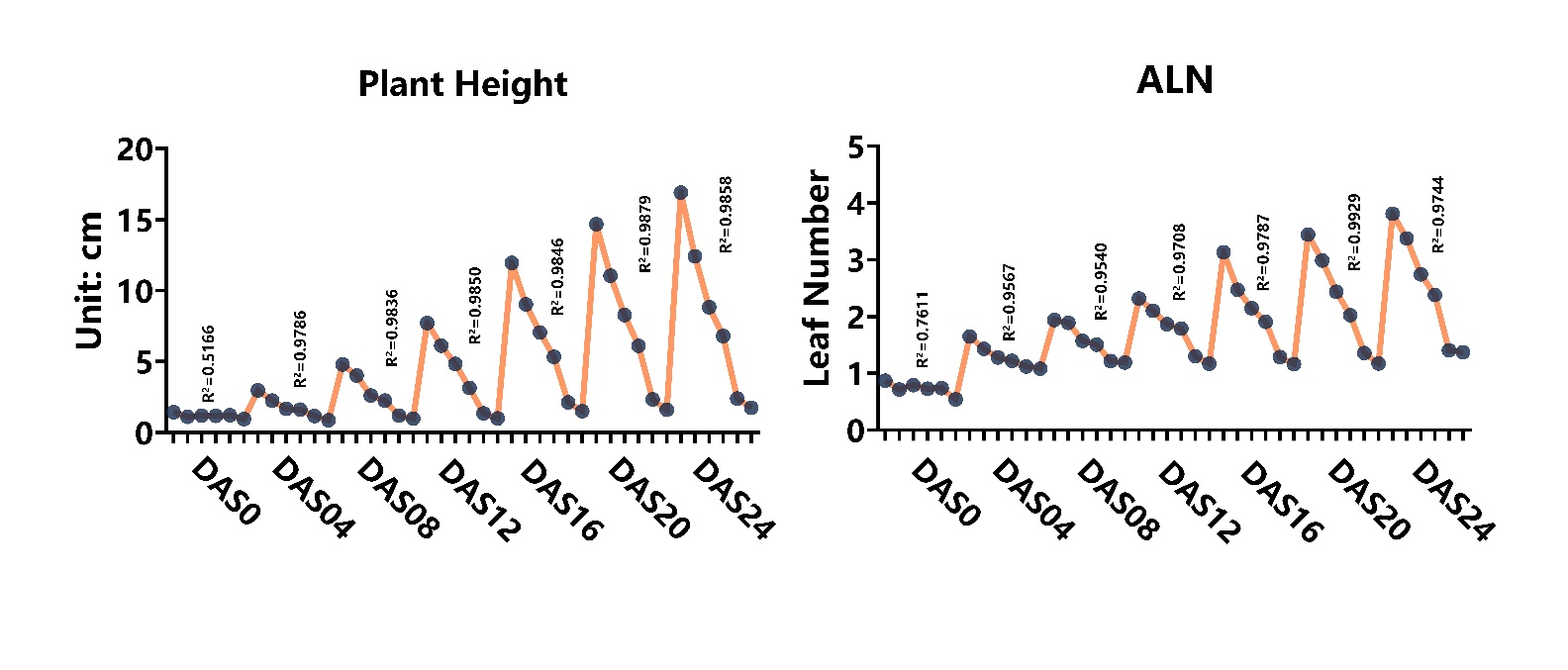


**Additional file 3. Presentation of morphological traits under multiple salinities.** Cv. Adalayd was used to determine sufficient duration for seedling incubation under salt stress. The first unfolded leaf stage was taken as the initial point for salt stress. Plant height and average leaf number (ALN) data were collected on DAS04, DAS08, DAS12, DAS16, DAS20, and DAS24, and lines were drawn. Calculated by sigmoidal function, the simulation coefficients R^2^ of each group of datasets are labeled in the graphs.
