# Supplementary material for: Low-cost and reliable substrate-based phenotyping platform for screening salt tolerance of cutting propagation-dependent grass, paspalum vaginatum
Source: Plant Methods. 2024 Jun 19;20:94. doi: 10.1186/s13007-024-01225-z (PMC11186238; doi:10.1186/s13007-024-01225-z)
Supplement: Supplementary file 4 — Supplementary Material 4 [file 13007_2024_1225_MOESM4_ESM.docx]

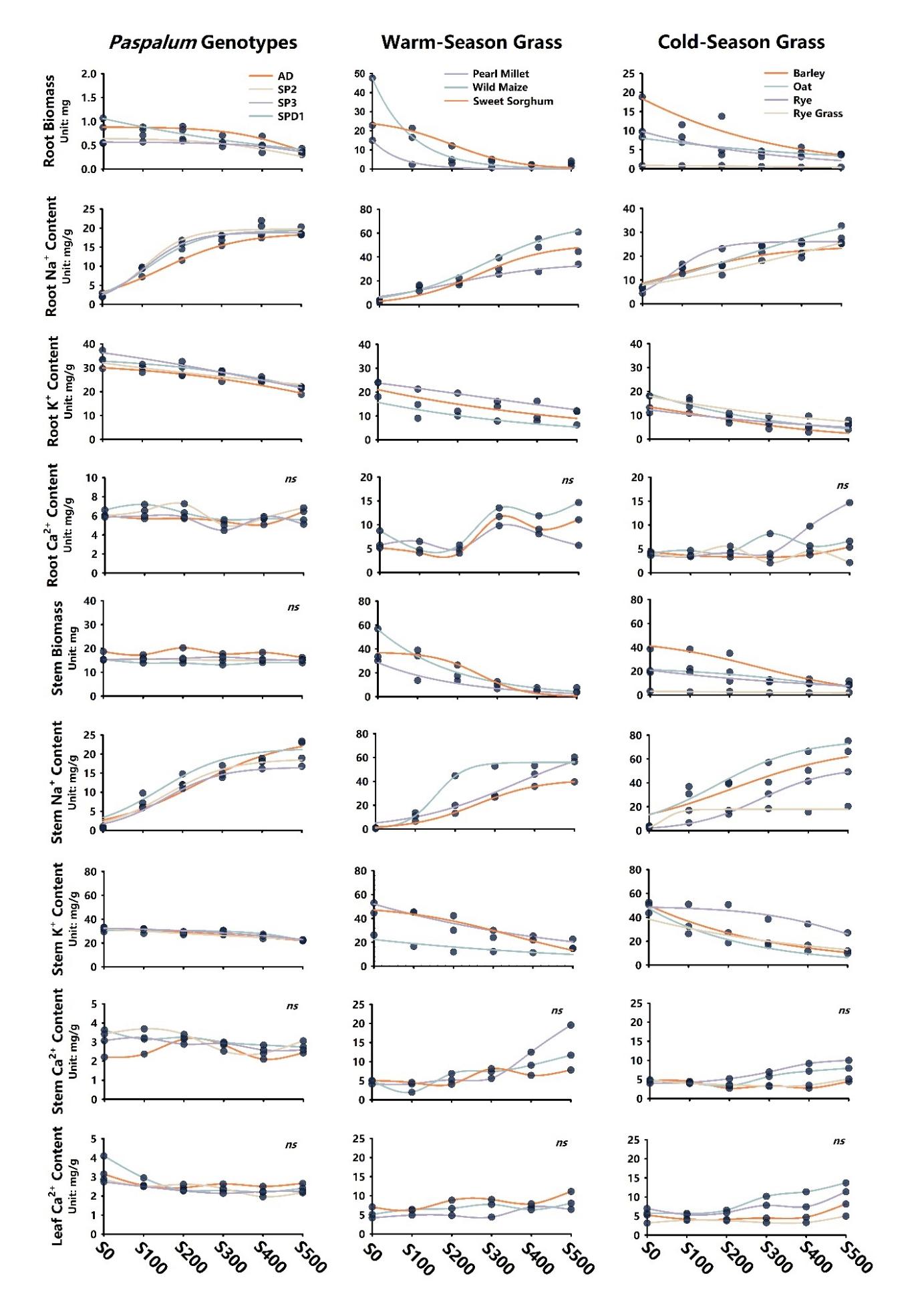


**Additional file 4. Additional trait dynamics among *P. vaginatum* genotypes, and warm-season and cold-season grass cultivars.** Biomass, Na^+^, K^+^, and Ca^2+^ contents in roots and stems are plotted against salinity level using the of 3-parameter Sigmoid function for the curve simulations. S0 = no salt; S100, S200, S300, S400, S500 = 100, 200, 300, 400 and 500 mM NaCl, respectively. Unsimulated traits are labeled “*ns*” and drawn by splines.
